# Bonding States of In Vitro Class 2 Direct Resin Composite Restoration Applied by Various Incremental Techniques

**DOI:** 10.3390/ma14206037

**Published:** 2021-10-13

**Authors:** Misato Okada, Masahiko Maeno, Yoichiro Nara

**Affiliations:** Department of Adhesive Dentistry, School of Life Dentistry at Tokyo, The Nippon Dental University, 1-9-20 Fujimi, Chiyoda-ku, Tokyo 102-8159, Japan; m-okada2118009@tky.ndu.ac.jp (M.O.); yo-nara@tky.ndu.ac.jp (Y.N.)

**Keywords:** bonding state, direct resin composite restoration, class 2 restoration, incremental technique, micro-tensile bond strength, cyclic loading, quantitative evaluation, qualitative evaluation, Weibull analysis

## Abstract

Incremental techniques are always required for clinical cases of deep and/or large cavities restored with resin composite materials. The purpose of this study was to examine the bonding states of class 2 direct resin composite restoration applied by various incremental techniques after cyclic loading to simulate the intra-oral environment to define the appropriate technique. Three types of resin composites, namely, bulk-fill (B), flowable (F), and conventional resin composite (C), were applied to standardized class 2 cavities by incremental techniques with single- or bi-resin restoratives. After cyclic loading, the micro-tensile bond strength (μ-TBS) of the dentin cavity floor was measured. The Weibull modulus and Weibull stress values at 10%/90% probability of failure were analyzed. Single-resin incremental restorations with B or F and bi-resin incremental restorations with F + B and F + C demonstrated superior μ-TBS (quantitative ability), bonding reliability, and durability (qualitative ability) compared with the single-resin restoration with C (as control). Furthermore, F + B and F + C restoration yielded an excellent performance compared with the single-resin restorations with B, F, and C. In particular, the F + C restoration, which indicates not only the maximum mean µ-TBS, but also the highest values of the Weibull parameters, may be the optimal restoration method, including the esthetic benefits.

## 1. Introduction

Minimally invasive and esthetic dental treatment based on the concept of minimal intervention dentistry (MID) [1] is a major patient desire throughout the world, regardless of age, gender, occupation, and nationality. In particular, direct restoration using resin composite restorative materials with resin adhesive systems is a typical treatment method used to realize the concept of MID because the treatment contributes to conservation of sound tooth substance and ensuring esthetics. Present resin composite restorative materials (physical properties, handling, and esthetics) have been improved considerably during the past decades and can be applied to various clinical cases. Hybrid resin composites, which have excellent mechanical properties and superior color-matching characteristics, can be used in a wide range of clinical cases [2]. Hybrid resin composites are positioned as conventional resin composite materials [3,4,5,6,7,8] and are popular and frequently used throughout the world. In recent hybrid resin composites, nanofilled resin composites have achieved high-mechanical properties and excellent operability because they contain a high concentration of nanoparticles [9].

Flowable resin composites, which exhibit outstanding flowability, wettability, and handling, have been employed in recent clinical practice [2]. First-generation flowable resin composites are not recommended for use in regions exposed to high stress, such as the occlusal surfaces and cusps of molars, anterior incisal edges, and canine cuspids, because of their poor mechanical properties [10]. However, recent flowable resin composites can be applied to medium and large class 2 cavities owing to their physical properties [11]. Incremental techniques are required for deep and/or large cavities with conventional and flowable resin composites [12]. Horizontal or oblique incremental techniques are conducted to reduce the polymerization shrinkage of resin composites filled in the cavity to achieve a good clinical prognosis [13,14]. In addition, it has been reported that the incremental technique increases the bond strength to the cavity floor [15]. Most resin composite materials require a 20 s light-curing time period based on the use of a well-conditioned light source, mean light irradiation of 1000 mW/cm^2^ from an 8 to 10 mm diameter light guide tip to adequately polymerize resin component increments with thicknesses from 1.5 to 2 mm [2,12]. Conversely, bulk-fill resin composites, which allow bulk filling to the deep cavity, have been introduced as a useful restorative material that can be applied in thick layers (thicknesses up to 4 mm), and save chair-time in comparison with previous incremental techniques [2,16,17,18]. Clarifications regarding the actual bonding states of direct resin composite restorations with single- or bi-resin restoratives after cyclic loading simulating the intra-oral environment will contribute to the determination of the appropriate restoration methods in clinical practice.

There are several types of class 2 complex cavities, for example, the mesial and occlusal surfaces (MO), the occlusal and distal surfaces (OD), and the mesial, occlusal and distal surfaces (MOD) cavities. MO cavities are associated with few technical limitations and are often treated by direct resin composite restorations that achieve esthetic treatment based on the MID concept.

The purpose of this study was to examine the bonding states of class 2 direct resin composite restorations applied according to five types of incremental technique: three types of single-resin incremental restoration with bulk-fill (B), flowable (F) and conventional resin composite (C) and two types of bi-resin incremental restorations with F + B (combined F and B) and F + C (combined F and C) after cyclic loading to simulate the intra-oral environment to identify the most appropriate technique. Based on the micro-tensile bond strength (µ-TBS) of five different incremental restorations, the bonding states were evaluated quantitatively and qualitatively.

The null hypothesis of this study is as follows: (1) five types of incremental restoration with the combination of resin composite restorative and incremental techniques do not influence the intra-cavity µ-TBS of class 2 direct resin composite restoration after cyclic loading, (2) the bonding reliability and durability of the resin composite restorations are not affected by the type of incremental restoration.

## 2. Materials and Methods

### 2.1. Experimental Material

The product name (shade), composition, lot number, and manufacturer of each material used in this study are listed in Table 1. For the restorative materials, three types of resin composite usable for class 2 direct restorations were selected: a bulk-fill resin composite (B: Tetric N-Ceram Bulk Fill, Ivoclar vivadent, Schaan, Liechtenstein), a flowable resin composite (F: G-aenial Universal Injectable, GC, Tokyo, Japan), and a conventional resin composite (C: Filtek Supreme Ultra Universal Restorative, 3M, St. Paul, MN, USA). An all-in-one adhesive system (Prime & Bond universal, Dentsply Sirona, York, PA, USA) was used as self-etch mode [19] for tooth surface treatment prior to all restorations using the three types of resin composite. For light irradiation, a light-emitting diode (LED) source (G-Light Prima II, GC) was used in the normal mode. Before and after each irradiation, the light intensity was measured using a radiometer (Demetron, L.E.D. Radiometer, Kerr, Orange, CA, USA), and the mean value of the output was 1560 mW/cm^2^ was confirmed.

### 2.2. Tooth Selection and Experimental Procedures

**Figure 1:**
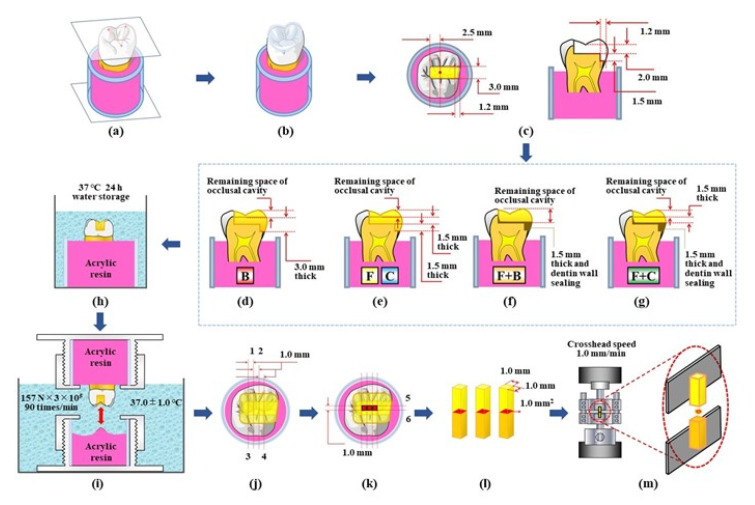
Flowchart of the experimental procedures. (**a**): Standardized tooth embedding, (**b**): Preparing of transparent occlusal shape stent, (**c**): Standardized cavity preparation, (**d**): B-restoration using a two-layered incremental technique with a single resin restorative, B, (**e**): F- and C-restorations using a three-layered incremental technique with a single resin restorative, F or C, (**f**): F + B-restoration using a two-layered incremental technique with the F and B resin restoratives, (**g**): F + C-restoration using a three-layered incremental technique with the F and C resin restoratives, (**h**): Water storage at 37 °C for 24 h, (**i**): Cyclic loading of 157 N at 90 cycles/min for 3 × 10^5^ cycles in water at 37 °C, (**j**–**l**): Sectioning in order to obtain beam test specimens for the µ-TBS test, (**m**): μ-TBS measurement at a crosshead speed of 1.0 mm/min.

This study was approved by the ethics committee of the Nippon Dental University, School of Life Dentistry at Tokyo for the use of extracted human molars (approval number: NDU-T2019-32). Thirty intact maxillary human molars with similar sizes and color tones, which have been stored in 0.1% thymol solution at 23 ± 2 °C for less than 1 year after the extraction, were used. A flowchart of the experimental procedures is shown in Figure 1.

Each tooth was embedded in a standardized cylindrical mold and an acrylic resin (PROVINICE, Shofu, Kyoto, Japan) to establish a plane, which was set by the three apexes of the buccomesial, buccodistal, and mesiopalatal cusps, parallel to the base plane of the mold (Figure 1a). An occlusal shape stent for each molar was prepared using a transparent flowable resin composite (Palfique Clear, Tokuyama Dental, Tokyo, Japan) (Figure 1b). A straight cylinder diamond bur (FG114, ISO 158 083 014, mean grid size: 100 µm, Shofu) was equipped in a custom-made cavity duplicator (Tokyo Giken, Tokyo, Japan) and used for the standardized cavity preparation. The burs were changed every five cavity preparations. To prepare a standardized MO class 2 cavity, the cavity was prepared first at a depth of 2.0 mm from the deepest part of the central fossa, with a buccolingual width of 3.0 mm, and a distal wall presented 2.5 mm away from the central fossa. The mesioproximal box was then prepared with a depth of 1.5 mm from the prepared pulpal wall which had a gingival wall with a width of 1.2 mm (Figure 1c). The standardized cavity specimens were randomly divided in five groups: three types of single-resin incremental restoration with B, F, and C, and two types of bi-resin incremental restoration with F + B and F + C. Single-resin incremental restoration using the conventional resin composite (C-restoration), which is the most basic and general method in clinical applications, was set as a control condition. Prior to each restoration, transparent polyester strips (Matrix tape 1939, 3M) were placed with a Tofflemire matrix retainer on each cavity specimen. Cavity surfaces were treated with an all-in-one adhesive system according to the manufacturer’s instructions. B-restoration was performed using a two-layered incremental technique with a single resin restorative, B. The first layer was horizontally applied to both the mesioproximal box space and occlusal cavity space (thickness of 3.0 mm from the gingival wall). In the second layer, the remaining space of the occlusal cavity was filled and formed with a transparent occlusal stent to reproduce the original crown form (Figure 1d). F- and C-restorations were conducted based on the use of a three-layered incremental technique with a single resin restorative, F or C. The first layer was applied to the mesioproximal box space (thickness of 1.5 mm from the gingival wall). The second layer was horizontally applied to the occlusal cavity space at a thickness of 1.5 mm from the pulpal wall. In the third layer, the remaining space of the occlusal cavity was filled and formed with an occlusal stent to reproduce the original crown form (Figure 1e). F + B-restoration was conducted using a two-layered incremental technique with the F and B resin restoratives. In the first layer, the mesioproximal box space was filled with F (thickness of 1.5 mm from the gingival wall). At the same time, the portion of dentin walls in the occlusal cavity was sealed with the restorative material, F. In the second layer, the remaining space of the cavity was filled with B and was formed with an occlusal stent to reproduce the original crown form (Figure 1f). F + C-restoration was conducted using a three-layered incremental technique with the F and C resin restoratives. The first layer was applied in the same manner as that for the F + B-restoration, the mesioproximal box space was filled with F, and the dentin walls of the occlusal cavity were simultaneously sealed with the restorative, F. The second layer was horizontally applied to the cavity space with C (thickness of 1.5 mm from the surface of the first layer). In the third layer, the remaining space of the cavity was filled with C and formed with an occlusal stent to reproduce the original crown form (Figure 1g). Every incremental layer was light-cured for 20 s regardless of the incremental restorations. All restored specimens were finished with a flame-type diamond bur (DP-04, Kuraray Noritake Dental, Tokyo, Japan), and then stored in water at 37 °C for 24 h (Figure 1h). Subsequently, each specimen was polished clinically with a shell-type diamond polisher (Compomaster, Shofu) for pits and fissures, and with a series of polishing discs (Sof-Lex XT, 3M) for smooth surfaces.

### 2.3. Cyclic Loading and Micro-Tensile Bond Strength Test

For each restored specimen, an opposing object was prepared as a receptor that was subjected to cyclic load stress by the inner inclined occlusal surfaces of the restored specimen using an acrylic resin (PROVINICE, Shofu) filled in a standardized mold. All restored specimens were subjected to a cyclic loading of 157 N at 90 cycles/min for 3 × 10^5^ cycles in water at 37 °C using a custom-made multifunction apparatus (Tokyo Giken) (Figure 1i). After cyclic loading, each restored specimen was sectioned six times with a diamond wire saw (DMS3400, Meiwafosis, Tokyo, Japan) to obtain three beam test specimens for the µ-TBS test. First, each restored specimen was sectioned buccopalatally twice such that the first sectioning was performed at 0.5 mm distally from the central fossa (1); the second cutting was performed at 0.5 mm mesially from the fossa (2), the third cutting was conducted at 1.0 mm distally from the first sectioned position (3), and the fourth cutting was performed at 1.0 mm mesially from the second cut position (4) (Figure 1j). Subsequently, the specimen was cut mesiodistally twice at 0.5 mm buccally (5) and palatally (6) from the central fossa, which were parallel to the buccal wall (Figure 1k). After the above sectioning steps, three standardized beam test specimens were obtained from each restored specimen (Figure 1l). The μ-TBS of each test specimen was measured at a crosshead speed of 1.0 mm/min based on the use of a universal testing machine (Autograph AG-1, Shimadzu, Kyoto, Japan) (Figure 1m).

### 2.4. Statistical Analysis

First, one-way analysis of variance (ANOVA) was applied to clarify the influence of the three different positions, that is, mesial, central, and distal positions on the dentin cavity floor, on the µ-TBS of each restoration using spreadsheet software (Excel 2016 Windows, Microsoft, Redmond, WA, USA) at a 5% level of significance. The µ-TBS obtained from every restoration group (*n* = 18 each) was analyzed using ANOVA and Tukey’s honestly significant difference (HSD) test using the same spreadsheet software at a 5% level of significance. In addition, to evaluate the bonding reliability and durability, three typical Weibull parameters were analyzed with the same spreadsheet software with a level of significance set at 5% based on µ-TBS, Weibull modulus (Wm), and Weibull stress values at a probability of failure of 10%/90% (PF10/PF90).

### 2.5. Fracture Mode Observation

The fracture mode of each post-test specimen was observed by using an optical microscope (Measurescope MM-11, Nikon, Tokyo, Japan) at a 200× magnification. Furthermore, to confirm the composition of the fractured surface, the dentin-side surface of the representative specimen selected from each restoration group was osmium-coated and then observed using a scanning electron microscope (SEM, JSE-IT200, Hitachi, Tokyo, Japan) with an accelerating voltage of 10.0 kV.

### 2.6. Basic Physical Properties of Three Resin Composite Materials

The linear polymerization shrinkage, flexural strength, and flexural modulus of the three resin composites used in this study were measured. The linear polymerization shrinkage was measured using a noncontact diode-laser displacement sensor (HL-C105B-BK; SUNX, Kasugai, Japan, *n* = 5) with a measurement accuracy of 1.0 μm according to a method reported by Miyasaka et al. [20]. The flexural strength was measured using a three-point flexural strength test according to the International Organization for Standardization (ISO) 4049:2019 guidelines [21]. The flexural modulus was calculated from the slope of the stress–strain curve obtained from the flexural strength test. Data were analyzed using ANOVA and Tukey’s HSD test using spreadsheet software (Excel 2016 Windows, Microsoft) with a level of significance set at 5%.

## 3. Results

### 3.1. Differences in the Mean Values of µ-TBS among Five Types of Resin Restoration

Prior to the statistical examination of the differences in the mean values of µ-TBS among the five types of resin restoration, it was confirmed that the µ-TBS did not vary as a function of the difference of the three positions, that is, mesial, central, and distal, regardless of the incremental restorations.

**Figure 2:**
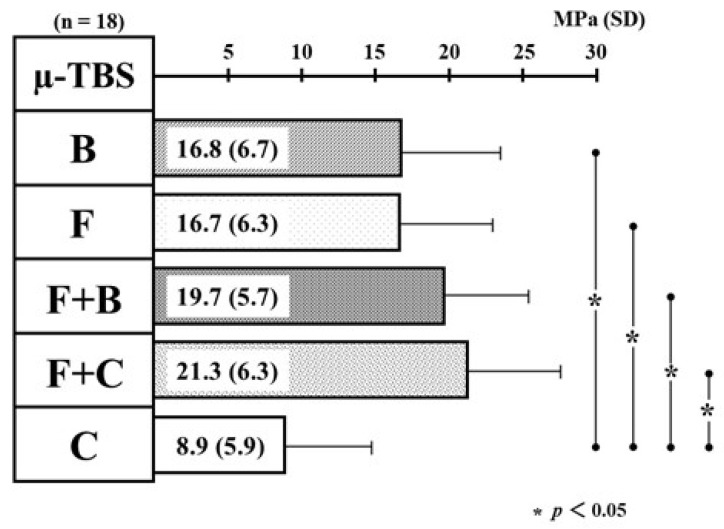
Differences in the mean value of micro-tensile bond strength among the five types of resin restoration.

Figure 2 shows the differences in the mean value of µ-TBS among the five types of resin restoration. The mean values of µ-TBS (SD) in B/F/F + B/F + C-restoration indicated 16.8 (6.7)/16.7 (6.3)/19.7 (5.7)/21.3 (6.3) MPa, and the values were 1.9/1.9/2.2/2.4 times statistically greater than the values of C-restoration [8.9 (5.9) MPa]. Therefore, single-resin incremental restoration with B and F and bi-resin incremental restorations with F + B and F + C demonstrated superior quantitative ability to obtain the bond strength compared with single-resin incremental restoration with C (as control).

### 3.2. Differences in the Values of Weibull Parameters among Five Types of Resin Restoration

**Figure 3:**
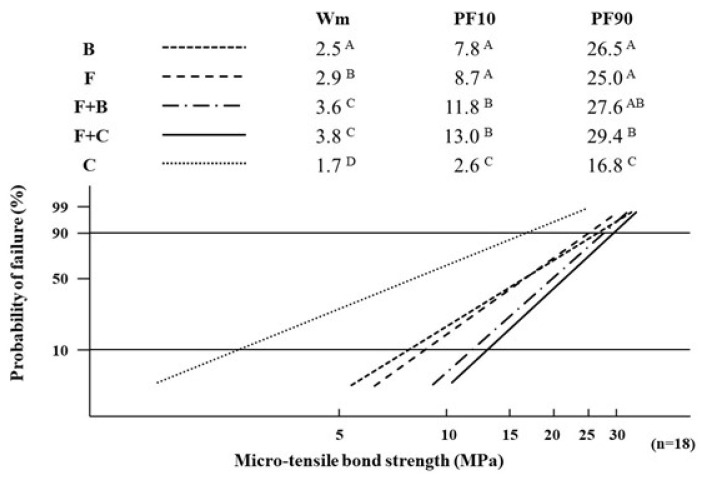
Differences in the values of Weibull parameters among the five types of resin restoration. Values with different letters in same column indicate a statistically significant difference at *p* < 0.05. Wm: Weibull modulus, PF10: Weibull stress (MPa) for a 10% failure probability, PF90: Weibull stress (MPa) for a 90% failure probability.

Figure 3 displays the differences in the values of the Weibull parameters, that is, Wm, PF10, and PF90, among the five types of resin restoration. For every Weibull parameter, the values of B/F/F + B/F + C-restoration were significantly greater than the value of C-restoration. The Wm, PF10, and PF90 of F + C-restoration showed the maximum value among the five types of resin restoration. Therefore, single-resin incremental restoration with B and F and bi-resin incremental restorations with F + B and F + C indicated superior bonding reliability and durability to single-resin incremental restoration with C (as control). The Wm of F-restoration was significantly greater than that of B-restoration, and there were no significant differences in PF10 and PF90 between the two restorations. Therefore, the bonding reliability of F-restoration was superior to that of B-restoration, but the bonding durability of F-restoration was similar to that of B-restoration. The Wm and PF10 of F + B-restoration, and the Wm, PF10, and PF90 of F + C-restoration were significantly greater than those of the B-, F-, and C-restorations. Moreover, there was no significant difference in the Weibull parameters between the F + B and F + C-restorations. From the above results, bi-resin incremental restorations with F + B and F + C where F was employed as the first-layered restorative, indicated superior bonding reliability and durability compared to single-resin incremental restoration with B, F, and C (as control). In particular, the F + C restoration supplied the most appropriate bonding states within the five types of resin restoration.

### 3.3. Fracture Mode Distribution of the Post-bond Test Specimens

Table 2 shows the fracture mode distributions of the post-bond test specimens.

The debonded surfaces of the post-bond test specimens consisted of three types of cohesive failure modes (Cf, Cs, Ca) and an interfacial failure mode (I). The cohesive failures observed on the debonded surfaces were composed of two types of fracture modes, Cf, within filling layer with each resin composite material (B, F, C) and Cs, within sealing layer with a flowable resin composite material (F). Interfacial failures occurred at the interface between the dentin and the filling or sealing layer. Mixed failures exhibited four types of fracture modes (CF + Cs, CF + Cs + Ca + I, Cs + Ca + I, and CF + Ca + I) which consisted of Cf, Cs, Ca, and I. The fracture mode of single-resin incremental restorations (B/F/C) was mainly mixed fracture, including I (61%, 61%, and 78%, respectively).

For the two types of bi-resin incremental restoration, 44% (eight specimens, the largest number) of all F + B specimens exhibited Cf, but F + C-restoration mainly showed mixed failure, including I (11 specimens, accounting for 61% of all specimens). Therefore, both restorations showed different tendencies in fracture mode distribution. Furthermore, for the bi-resin incremental restorations, the percentages of mixed failure including I for F + B/F + C-restorations were 33%/61%, and the value of F + B-restoration was remarkably smaller than that (78%) of the C-restoration. From the above, it was clarified that the fracture mode of the five types of class 2 direct resin composite restorations varied following the application of the resin composite restorative, and following the execution of the incremental technique.

**Figure 4:**
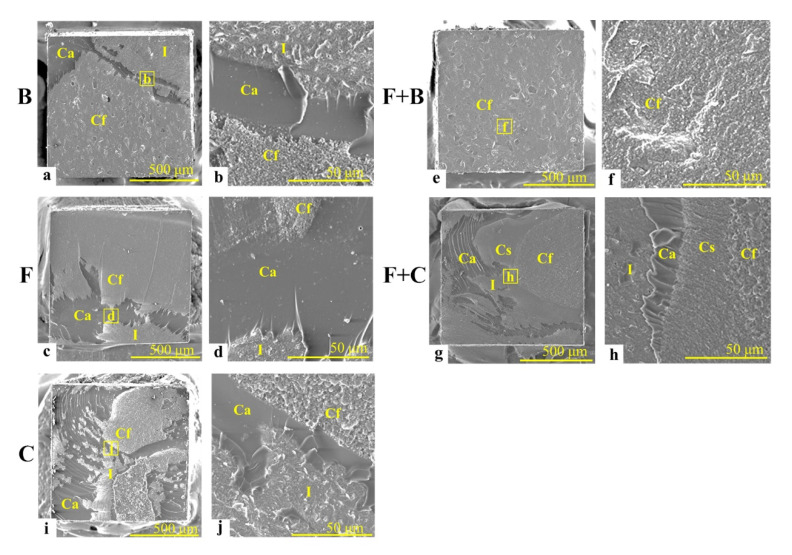
Representative scanning electron microscopy images (50× and 500× magnifications) of dentin-side surface of the post-test specimens. Cf: Cohesive failure occurred within filling layer with each resin composite material (B, F, C), Cs: Cohesive failure occurred within sealing layer with flowable resin composite material (F), Ca: Cohesive failure occurred within adhesive layer, I: Interfacial failure occurred at the interfaces between filling or sealing layer and dentin. (**a**,**b**): Low- and high-magnification image of B-restoration consisted of I, Ca, and Cf, (**c**,**d**): Low- and high-magnification image of F-restoration consisted of I, Ca, and Cf, (**e**,**f**): Low- and high-magnification image of F + B-restoration consisted of Cf, (**g**,**h**): Low- and high-magnification image of F + C-restoration consisted of I, Ca, Cs, and Cf, (**i**,**j**): Low- and high-magnification image of C-restoration consisted of I, Ca, and Cf.

Figure 4 shows representative SEM images (50× and 500× magnifications) of the dentin-side surface of the post-bond test specimens.

In the cases of the B/F/C/F + C-restoration, cohesive failure occurred within adhesive layer (Ca) at the boundary areas between the interfacial failure (I) and cohesive failure parts which occurred within the filling layer (Cf). In the low-magnification image of B-restoration, the mixed failure consisted of a particulate flattened surface (I), a smooth surface with minute corrugations in some places (Ca), and a flattened surface with paisley-patterns (Cf) was observed (Figure 4a). In the high-magnification image of B-restoration, the portion of Cf displayed the granular-patterned surface of the resin composite restorative (B) (Figure 4b). In the low-magnification image of F-restoration, the mixed failure consisted of I and Ca surfaces similar to those of B-restoration at Figure 4a, and smooth and flattened surfaces with minute corrugations in some places (Cf) were recognized (Figure 4c). In the high-magnification image of F-restoration, the part of Cf showed a granular-patterned surface of the resin composite restorative (F) (Figure 4d). In the low-magnification image of C-restoration, the mixed failure consisted of I and Ca surfaces similar with those of B- and F-restorations at Figure 4a,c, and a coarse flattened surface (Cf) was recognized (Figure 4i). In the high-magnification image of C-restoration (as control), a relatively larger granular-patterned surface of the resin composite restorative (C) was observed (Figure 4j). Conversely, in both the low and high-magnification images of F + B-restoration, the entire Cf surface was identified similar to the Cf images of B-restoration (Figure 4e,f). In the low-magnification image of F + C-restoration, the mixed failure consisted of I and Ca surfaces similar with those of the B-, F-, and C-restorations at Figure 4a,c,e, a Cs surface similar with the Cf image observed in F-restoration; accordingly, a Cf surface looking like the Cf image of C-restoration is indicated (Figure 4g). In the high-magnification image of the F + C-restoration, a Cs surface similar to the Cf image observed in F-restoration and Cf surface resembling the Cf image of C-restoration was identified (Figure 4h). Furthermore, at the high-magnification I-images of B/F/C/F + C-restoration, dentinal tubules sealed by the adhesive were observed.

### 3.4. Basic Properties of Three Resin Composite Materials

Table 3 displays the mean values of the linear polymerization shrinkage, flexural strength, and flexural modulus of the three resin composite materials. For the linear polymerization shrinkage, the F value was significantly greater than those of C (as control) and B. For the flexural strength, the B value was significantly smaller than those of C (as control) and F. For the flexural modulus, the value decreased in the order of C > B > F, and significant differences were observed between any two materials.

## 4. Discussion

### 4.1. Differences in the Mean Values of µ-TBS among Five Types of Resin Restoration

Based on the results from the mean values of µ-TBS, B/F/F + B/F + C-restoration demonstrated excellent ability to achieve high-bond strength compared with C-restoration (as control). This study was conducted subject to an experimental dynamic stress condition of 157 N loading at 90 cycles/min. The condition was set based on previous studies that described the stress of human mastication, which was in the range of 70–150 N [22,23]; the average number of mastication per minute was in the range of 60–90 times [24,25]. The total number of cyclic loadings in this study (3 × 10^5^ times) corresponds to 14 months according to a report in which the average number of human mastication per year was approximately 2.5 × 10^5^ [26]. The condition was severe because it was conducted continually without any rest and reproduced a rugged mastication environment (e.g., during the entire day without sleep or rest for 14 months). The bond strength of restorations exposed to such severe cyclic loading should be influenced by the basic properties of the restorative materials used.

First, focusing on three types of single-resin incremental restoration, the bond strengths of both B- and F-restorations were significantly greater than the value of C-restoration. The linear polymerization shrinkage (Table 3) of F was significantly greater than those of B and C (as control). The effect of restoration with a resin composite with a high-polymerization shrinkage on the adhesive interface was greater than that of restorations with resin composites having low shrinkage values. However, the bond strength of the F-restoration was significantly greater than that of the C-restoration and was not statistically different from the value of B-restoration. Therefore, the effect of linear polymerization shrinkage on the bond strength may be weakened by the application of the incremental technique and other properties of resin composites. The flexural strength (Table 3) of B was significantly lower than those of F and C. The flexural strength of a resin composite is an important indicator of fracture resistance and may be correlated with the occurrence of marginal or body fractures by mastication. It is inferred that there is no clear relationship between the flexural strength and bond strength in this study. The flexural moduli (Table 3) of F and B were significantly smaller than those of C, and F indicated a significantly smaller modulus than B. Therefore, it is clarified that the flexural modulus clearly influences the bond strength of class 2 direct resin composite restorations compared with the linear polymerization shrinkage and flexural strength. Ishii et al. [27] reported that the transformation of restoration itself absorbs cyclic loading and reduces the risk of bonding failure in the case of restorations with materials which have a small flexural modulus. For single-resin incremental restorations, it can be considered that B and F, which have significantly smaller flexural moduli than C, produced plastic deformation and acted as a stress breaker against cyclic loading. This reduced the damage to the bonding of the cavity floor. Based on the results presented above, in terms of quantitative bonding, it was confirmed that the single-resin incremental restorations with B or F were superior to the restoration with C.

However, in terms of esthetic restoration, B which was developed with a focus on light transmission to obtain deep curing-depth, was limited to select color tone compared with C which provides various color tones. Therefore, especially for resin restoration in the region requiring esthetics, the single-resin incremental restoration with B makes it difficult to achieve proper color matching with natural teeth. Conversely, Shinkai et al. [28] reported that increased surface degradation, which was observed in the form of radial cracks, was observed on the surfaces of flowable resin composites subjected to cyclic loading compared with the loaded surface of a conventional resin composite. Accordingly, the single-resin incremental restoration with F applied to the occlusal area may increase the risk of material failure. From the above, in clinical practice, it seems that single-resin incremental restorations are not able to achieve sufficient clinical results desired by both patients and dentists, that is, results manifested by robust bonding, esthetic recovery, and reduced risk of poor prognosis.

Focusing on bi-resin incremental restorations, the mean µ-TBS values of F + B and F + C-restorations were two times greater than those of C-restoration (as control). In the bi-resin incremental restorations, the mesioproximal box space was filled with F, and the portion of dentin walls in the occlusal cavity was film-likely sealed with the restorative. In the flexural modulus (Table 3), the value of F (6.3 GPa) was the lowest, that of C (11.7 GPa) was the largest, and the B value (8.1 GPa) was moderate; additionally, there were significant differences among the three resin composites. Therefore, it can be considered that the existence of F, which indicates the lowest flexural modulus and acts as a stress breaker for both F + B and F + C-restorations, contributes to the higher bond strengths compared with C-restoration (as control) such that the stress of cyclic loading directly transmits to the adhesive interface because of the large flexural modulus of C.

### 4.2. Differences in the Values of Weibull Parameters among Five Types of Resin Restoration

In this study, the µ-TBS values to the internal dentin cavity floor of restored teeth were measured, and the quantitative evaluation for the µ-TBS using ANOVA and Tukey’s HSD test, and the qualitative evaluation of the bonding reliability and durability using Weibull analysis [29] were conducted. In particular, ISO [30] states that the Weibull stress values for a 10% and 90% failure probability level (PF10 and PF90) are convenient ways to characterize the strength of a bond. In addition, a large Weibull modulus (Wm) was preferable because it indicates an improved homogeneity in the flaw population and a high prediction for failure behavior, regardless of the material [31]. It was also reported that a large value of Wm indicated high-bonding reliability [32].

The results of the Weibull analysis showed that single-resin incremental restorations with B or F and bi-resin incremental restorations with F + B and F + C demonstrated superior performance to single-resin incremental restoration with C (as control). From the above, it is confirmed by the qualitative evaluation that the positive effects, based on the stress breaker function with the small flexural modulus values of B and F in the single-resin incremental restorations and the absorbing function of the sealing layer on the cavity floor using F in bi-resin incremental restorations with F + B and F + C, are very effective for improving the bonding states of resin restorations. In addition, there was no significant difference in the quantitative evaluation based on the µ-TBS between the single-resin incremental restorations with B or F, although F-restoration indicated a significantly better bonding reliability than B-restoration based on the Wm values from the qualitative evaluation. Kawai et al. [33] evaluated the bonding of flowable resin composites after cyclic loading compared with a conventional resin composite (as control). There are some reports on the basic evaluation of bulk-fill resin composites, such as mechanical properties and fracture reliability [34,35], fracture strength, and polymerization shrinkage stress [36]. However, no study has examined the bonding state of bulk-fill resin composite restorations under cyclic loading conditions simulating the intra-oral environment. In the basic properties of resin composites used in this study (Table 3), the flexural strength of F (180.8 MPa) was significantly greater than the value of B (131.0 MPa), and the flexural modulus of F (6.3 GPa) was significantly smaller than that of B (8.1 GPa). Therefore, it can be regarded that F is superior in the resistance against the fracture caused by the external force to B and acts as a stress breaker against the force. Furthermore, the linear polymerization shrinkage of F (2.3%) was greater than that of B (1.7%). The positive effect of F-restoration with a large polymerization shrinkage on the bonding reliability should be theoretically smaller than B. For the incremental technique, three and two incremental techniques were applied to F and B restorations, respectively. The positive effect of F-restoration with three increments on the bonding reliability should theoretically be greater than that of B-restoration with two increments. From the results of the Wm value (Figure 3), the incremental technique may contribute to better bonding reliability compared with the linear polymerization shrinkage of the resin composite used. From the above, it can be considered that the bonding reliability of F-restoration is superior to B-restoration by the combined effect of the fracture resistance based on the flexural strength of resin composites, the stress breaker function based on the flexural modulus, and the number of incremental layers, which reduces the negative effect of linear polymerization shrinkage.

The bi-resin incremental restorations with F + B or F + C, which employed F as the first layer, showed excellent bonding reliability, and durability, compared with single-resin incremental restorations with B, F, and C (as control). This result indicates that the bi-resin incremental restorations, which provide both the absorbing effect by the sealing layer on the cavity floor acting as the stress breaker function and shrinkage-reducing effect by the incremental technique, can be expected to achieve a high-quality bonding state in clinical practice compared with single-resin incremental restorations. The polymerization shrinkage stress occurring at the bonded interface increases or decreases in proportion to the C-value. In most of the C-values in clinical situations, the stress-releasing flow is not sufficient to maintain adhesion to dentin by the applied resin adhesive systems [37]. For the incremental technique, it seemed that the C-value of the first layer applied to the dentin cavity walls influenced the bond strength, bonding reliability, and durability of the resin composite restoration process. The part of µ-TBS measured in this study was set at the center of the pulpal wall, including the area underneath the central fossa. Therefore, the C-values of the first layer in B-restoration, the second layer in both F and C-restorations, and the sealing layer on the pulpal dentin wall of the first layer in both F + B and F + C-restorations may influence the bonding reliability and durability of each restoration. The single-resin incremental restorations with B, F, and C were filled with each resin material (thickness of 1.5 mm) from the pulpal dentin wall and were then light-cured. Conversely, for the bi-resin incremental restorations with F + B and F + C, all intra-cavity dentin walls were film-likely sealed with F at a standardized 0.1 mm thickness, and then light-cured. The C-values on the pulpal dentin wall of the first layer of B-restoration, the second layer of F- and C-restorations, and the sealing part of the bi-resin incremental restorations with F + B and F + C were 1.82, 1.82, and 1.20, respectively. Therefore, the negative effect of the bi-resin incremental restorations with a thin sealing layer using F on the bonding to the dentin cavity floor should be weaker than that of the single-resin incremental restorations because of the polymerization shrinkage stress occurring on the cavity floor. Consequently, it is inferred that the bi-resin incremental restorations demonstrate excellent bonding reliability and durability compared with single-resin incremental restorations.

All Weibull parameters of F + C-restoration indicated the highest values among the five types of resin restoration, and the restoration supplied the most appropriate bonding states among the five types of restoration. For esthetic restoration, C is a sufficient restorative material applied to the superficial occlusal part of F + C-restoration because of the variety of options for color tones. Based on the above, the F + C-restoration, which indicates not only the maximum mean µ-TBS as a quantitative evaluation result, but also the highest values of the Weibull parameters as the qualitative evaluation results, may be the optimal restoration method, including the esthetic benefits.

### 4.3. Fracture Mode Distribution of the Measured Specimens

The fracture modes in the single-resin incremental restorations with B, F, and C were mainly mixed fractures, including I. This fact suggests that the incremental, single-resin restorations with resin composites having different basic properties influence the results of quantitative and qualitative evaluations, but the fracture mode distribution does not vary with the incremental restorations. Conversely, for the bi-resin incremental restorations with the F + B, Cf-mode (cohesive failure within B) was frequently observed. The Cf which occurred within B can be attributed to the fact that the flexural strength of the B value was significantly smaller than that of F (Table 3). Furthermore, for the bi-resin incremental restorations, the F + C-restoration had a higher percentage of mixed fractures (including I) than that of F + B-restoration. The flexural modulus of C was significantly greater than those of B and F (Table 3). The material with a high flexural modulus demonstrates a low strain compared with a material with a low modulus [16]. Therefore, a higher percentage of mixed fractures including I in the F + C-restoration in comparison with the F + B-restoration may be caused by the transferred stress that was not absorbed in C but passed through the material because of its high flexural modulus and damaged the interface between the F-sealing layer and dentin. A similar phenomenon was confirmed at a very high percentage (78%) of mixed fractures, including I in C-restoration.

## 5. Conclusions

Within the limitations of the present study, the conclusions are as follows:

(1) Single-resin incremental restorations with a bulk-fill resin composite (B) or a flowable resin composite (F) and bi-resin incremental restorations with F + B and F + C demonstrated superior quantitative and qualitative abilities in achieving the bond strength, bonding reliability, and durability compared with single-resin incremental restorations with the conventional resin composite C (as control)
(2) The bi-resin incremental restorations with F + B and F + C, where F was employed as the first-layered restorative, indicated excellent performance in bonding reliability and durability compared with the single-resin incremental restorations with B, F, and C
(3) F + C-restoration, which indicates not only the maximum mean µ-TBS, but also the highest values of the Weibull parameters, supplied the most appropriate bonding states within the five types of resin restoration.

## Figures and Tables

**Table 1:** Materials used in this study.

Resin Composite Material		Composition	Lot Number	Manufacturer
Bulk-Fill resin composite [Code: B]	Tetric N-Ceram Bulk Fill (Shade: IVA)	Barium glass filler, Bis-EMA, Bis-GMA, UDMA, ytterbium trifluoride	Z001JF	Ivoclar vivadent
Flowable resin composite [Code: F]	G-aenial Universal Injectable (Shade: A2)	Barium glass filler, Bis-MEPP, Bis-EMA, bismethacrylate, dimethacrylate, UV-light absorber, UDMA, dimethacrylate component	2003161	GC
Conventional resin composite [Code: C]	Filtek Supreme Ultra Universal Restorative (Shade: A2)	Silane treated ceramic, Bis-EMA, Bis-GMA, UDMA, Silane treated silica, PEGDMA, Silane treated zirconia, Triethylene glycol dimethacrylate	NC00725	3M
**Resin Adhesive System**		**Composition**	**Lot Number**	**Manufacturer**
All-in-one adhesive system	Prime & Bond universal	Phosphoric acid modified acrylate resin, Initiaor, Stabilizer, Multifunctional acrylate, Isopropanol, Bifunctional acrylate, Acidic acrylate, Water	1909000418	Dentsply Sirona

Bis-EMA: bisphenol A polyethethylene glycol diether dimethacrylate, Bis-GMA: bisphenol A glycidyl methacrylate, UDMA: urethane dimethacrylate, Bis-MEPP: 2,2-bis-(4-methacryloxyphenyl)propane, PEGDMA: polyethylene glycol dimethacrylate.

**Table 2:** Fracture mode distribution of the post-bond test specimens.

Classification	Fracture Mode Composition	Five Types of Class 2 Resin Composite Restoration				
		B	F	F + B	F + C	C
Cohesive failure	Cf	6	7	8	5	4
	Cs	—	—	0	0	—
Interfacial failure	I	1	0	0	0	0
Mixed failure	CF + Cs	—	—	4	2	—
	CF + Cs + Ca + I	—	—	4	7	—
	Cs + Ca + I	—	—	2	4	—
	CF + Ca + I	11	11	—	—	14

Cf: Cohesive failure occurred within filling layer with each resin composite material (B, F, C); Cs: Cohesive failure occurred within sealing layer with flowable resin composite material (F); Ca: Cohesive failure occurred within adhesive layer; I: Interfacial failure occurred at the interfaces between between filling or sealing layer and dentin.

**Table 3:** Mean values of linear polymerization shrinkage, flexural strength and flexural modulus of three resin composite materials.

Resin Composite Material	Linear Polymerization Shrinkage (%)	Flexural Strength (MPa)	Mean Value (SD) (*n* = 5)
			Flexural Modulus (GPa)
B	1.7 (0.3) ^A^	131.0 (11.7) ^A^	8.1 (0.9) ^A^
F	2.3 (0.2) ^B^	180.8 (22.6) ^B^	6.3 (0.9) ^B^
C	1.6 (0.3) ^A^	186.5 (18.1) ^B^	11.7 (0.9) ^C^

Values with different letters (A, B, C) in same column indicate a statistically significant difference at *p* < 0.05.

## Data Availability

Data available on request due to restrictions. The data presented in this study are available on request from the corresponding author.
